# Molecular Weight Enables Fine-Tuning the Thermal and Dielectric Properties of Polymethacrylates Bearing Sulfonyl and Nitrile Groups as Dipolar Entities

**DOI:** 10.3390/polym13030317

**Published:** 2021-01-20

**Authors:** Sebastian Bonardd, Cesar Saldías, Ángel Leiva, David Díaz Díaz, Galder Kortaberria

**Affiliations:** 1 Facultad de Ciencias, Centro de Nanotecnología Aplicada, Universidad Mayor, Camino la Pirámide 5750, Santiago 8580745, Chile; 2 Departamento de Química Física, Facultad de Química y de Farmacia, Pontificia Universidad Católica de Chile, Santiago 7820436, Chile; casaldia@uc.cl (C.S.); aleivac@uc.cl (Á.L.); 3 Departamento de Química Orgánica, Universidad de La Laguna, Avda, Astrofísico Francisco Sánchez S/N, 38206 La Laguna, Tenerife, Spain; ddiazdiaz@ull.edu.es; 4 Instituto Universitario de Bio-Orgánica Antonio González, Universidad de La Laguna, Avda, Astrofísico Francisco Sánchez 2, 38206 La Laguna, Tenerife, Spain; 5 Institut für Organische Chemie, Universität Regensburg, Universitätsstr. 31, 93053 Regensburg, Germany; 6 Universidad País Vasco/Euskal Herriko Unibertsitatea, ‘Materials + Technologies’ Group, Dpto. Ingeniería Química y Medio Ambiente, Escuela Univ Politécnica, Pza Europa 1, 20018 Donostia-San Sebastián, Gipuzkoa, Spain; galder.cortaberria@ehu.es

**Keywords:** polymer dielectrics, dipolar glass polymer, dielectric constant, loss tangent, energy storage

## Abstract

In this work, polymethacrylates containing sulfonyl and nitrile functional groups were successfully prepared by conventional radical polymerization and reversible addition-fragmentation chain-transfer polymerization (RAFT). The thermal and dielectric properties were evaluated, for the first time, considering differences in their molecular weights and dispersity values. Variations of the aforementioned properties do not seem to substantially affect the polarized state of these materials, defined in terms of the parameters ε’_r_, ε”_r_ and tan (δ). However, the earlier appearance of dissipative phenomena on the temperature scale for materials with lower molecular weights or broader molecular weight distributions, narrows the range of working temperatures in which they exhibit high dielectric constants along with low loss factors. Notwithstanding the above, as all polymers showed, at room temperature, ε’_r_ values above 9 and loss factors below 0.02, presenting higher dielectric performance when compared to conventional polymer materials, they could be considered as good candidates for energy storage applications.

## 1. Introduction

Since their first report carried out by Zhu and collaborators [1], *dipolar glass polymers* have served as inspiration for the development of numerous new polymer dielectrics exhibiting high dielectric constants and relatively low loss factors [2,3,4,5,6,7,8,9,10,11]. The outstanding dielectric properties shown by these materials have been related to the nature of their polarization process, which is mainly governed by the orientational mechanism [12]. This mechanism stands out over the others by achieving a high polarized state while keeping a relatively low dissipation behavior, since other mechanisms capable of reaching high polarizations (e.g., ionic and interfacial), usually depend on the presence of ionic species, whose diffusion increases the dissipative character of the material, in addition to affecting its much-needed insulating property [1,12,13]. To date, there is a significant number of reports that summarize and explain the physics and chemistry behind this class of materials, allowing to improve their understanding [12,13,14,15,16,17,18].

In brief, a *dipolar glass polymer* must fulfill two major requirements: (1) Being an amorphous material with the highest possible value for its glass transition temperature (*T*_g_) (2) having in its structure molecular entities with high and permanent dipole moments, capable of performing rotational movements at the lowest possible temperature, called Sub-*T*_g_ transitions [1,18]. The first requirement is based on the fact that amorphous materials exhibit higher free volume in their inner structure [19], facilitating the motions of dipolar structures [6]. On the other hand, a high *T*_g_ value avoids that highly dissipative phenomena, such as ionic conductivity, take place at the working temperatures of the material [7,13,20]. Regarding the second requirement, the use of sulfonyl and nitrile groups as dipole structures has proven to be a good strategy since, in addition to their high dipole moments (4.25 D and 3.9 D, respectively) [1], their movements begin to be active at temperatures well below room temperature [3,4,5,9,11,21,22,23,24,25]. In this way, polymers that meet the aforementioned requirements are able to exhibit a high polarized state over a wide range of temperatures before dissipative phenomena begin to occur.

In recent years, the growing interest in new all-polymer dielectric materials has been supported by the abrupt increase in the number of reports about *dipolar glass polymers* [3,4,5,6,7,8,10,15,18,20,21]. However, within this wide range of materials, those belonging to polymethacrylates family are considered as iconic examples. The above because the first dipolar glass polymer reported was the poly(2-(methylsulfonyl)ethyl methacrylate) (PSO_2_MA) prepared and characterized by Wei et al. [1] This polymer showed outstanding properties in terms of ε’_r_ and tan(δ), which recorded values at 1 Hz and room temperature were 11.4 and 0.02, respectively. Then, based on that pioneering work, our group reported for the first time the dielectric characterization for its structural analogue, the poly(2-cyanoethyl methacrylate) (PCNMA) [7]. The above work consisted in a complete comparison between both polymethacrylates, at the theoretical and experimental level, studying the effect of the dipolar group over their dielectric behavior but also their structural and thermal properties. However, in both works, these dipolar glass polymers were synthetized through conventional radical polymerization, achieving the obtention of polymer materials with high dispersities and poor reproducibility in terms of molecular weights. Indeed, by following the protocol reported by Wei et al. [1], our group have replicated, at two different opportunities (this work being one of them), the polymerization of PSO_2_MA, achieving on both occasions dissimilar results for the aforementioned parameters [7]. Thereby, and considering the advanced state of the polymer science regarding controlled polymerization methods [26], it is interesting to evaluate the possible effect that variations in molecular weights and dispersion values could have on the dielectric behavior of these polymers.

The present work aims to serve as motivation for the synthesis of new dielectric polymers through the use of controlled polymerization techniques, in order to better define their behavior, properties, and potential applications. Reversible addition-fragmentation chain-transfer polymerization (RAFT) polymerization was chosen mainly because it has been shown to be compatible with a wider range of monomers and to have a simpler experimental protocol [27,28]. In addition, since no inorganic complexes are required as catalyst, we avoid the introduction of ionic species in the final material. Both polymethacrylates were successfully prepared by conventional radical polymerization and by RAFT. The structural characterization was carried out by means of infrared spectroscopy (FTIR), ^1^H and ^13^C-NMR, whereas their molecular weights and dispersity values were measured by size-exclusion chromatography (SEC). The thermal characterization was achieved by thermogravimetric analysis (TGA) and differential scanning calorimetry (DSC), while dielectric properties were measured using broadband dielectric spectroscopy (BDS).

## 2. Experimental Part

### 2.1. Materials

Methacrylic acid (99%), benzoyl chloride (99%), 3-hydroxipropionitrile (99%), triethylamine (Et_3_N, 99%), dicyclohexylcarbodiimide (DCC, 98%), 4-dimethylaminopyridine (DMAP, 99%) and 2-cyano-2-propyl benzodithioate (2CPBz, >97%) were purchased from Sigma-Aldrich, while 2-(methylsulfonyl)ethanol was purchased from AK Scientific and azobis(isobutyronitrile) (AIBN) from Fluka. All solvents, purchased from Merck, were distilled prior to use.

### 2.2. Monomer Synthesis

The protocols followed for the synthesis of methacrylic monomers containing sulfones (SO_2_MA) or nitriles (CNMA) can be found in previous works reported by our group [7,20].

### 2.3. Polymer Synthesis

Both types of poly(methacrylate)s were synthesized through conventional radical polymerization and RAFT, following the protocols explained below.

#### 2.3.1. Conventional Radical Polymerization of Poly(2-cyanoethyl methacrylate) (PCNMA) and Poly(2-(methylsulfonyl)ethyl methacrylate) (PSO_2_MA)

The preparation of PCNMA and PSO_2_MA was achieved using the procedure reported by Wei et al. [1] with slight modifications. Two grams of monomer (SO_2_MA or CNMA) and a certain amount of AIBN (0.5 mol %) as a radical source were charged in a Schlenk tube equipped with a magnetic stirrer. Then, 10 mL of *N,N*-dimethylformamide (DMF) were added and stirred until the obtention of a clear and transparent solution. Five freeze-pump-thaws were performed, and the system was filled with dry N_2_ gas. The polymerization was started by heating the system at 70 °C under constant stirring and maintaining these conditions during 24 h, after which the polymerization mixture was poured over an excess of cold methanol inducing the apparition of a white powder. The polymeric powder was purified by three solubilization-precipitation cycles using DMF and methanol as solvent and precipitant, respectively. Obtained polymers were soaked in Milli-Q water for two days to remove DMF traces (changing water twice a day) for being then dried in a vacuum oven at 120 °C until constant weight. Polymerization conversions of 59 and 62% were obtained for PSO_2_MA and PCNMA, respectively.

#### 2.3.2. Reversible Addition-Fragmentation Chain-Transfer Polymerization of Poly(2-cyanoethyl methacrylate) (PCNMA-RAFT) and Poly(2-(methylsulfonyl)ethyl methacrylate) (PSO_2_MA-RAFT)

The synthesis of poly(methacrylate)s was carried out with AIBN as radical source and 2-cyano-2-propyl benzodithioate (2CPBz) as RAFT agent [29]. Briefly, for PCNMA-RAFT, 2.7 g of CNMA, 21.5 mg of 2CPBz, and 1.5 mg of AIBN were solubilized in 1 mL of DMF and placed in a Schlenk tube equipped with a magnetic stirrer. For poly(methacrylate)s containing sulfonyl groups, two samples of different molecular weight were prepared, labelled as PSO_2_MA-RAFT1 and PSO_2_MA-RAFT2. PSO_2_MA-RAFT1 was prepared from 2.0 g of SO_2_MA, 5.9 mg of 2CPBz, and 0.4 mg of AIBN, while PSO_2_MA-RAFT2 was prepared from 2.0 g of SO_2_MA, 13 mg of 2CPBz, and 1.3 mg of AIBN. In each case, reagents were solubilized in 1 mL of dioxane and placed in a Schlenk tube equipped with a magnetic stirrer. All pre-polymerization mixtures were subjected to five freeze-pump-thaws cycles and finally purged with dried N_2_. Polymerizations were carried out at 70 °C and under constant magnetic stirring for 24 h, after which each polymerization mixture was poured over an excess of cold methanol inducing the apparition of a pink powder. Polymers were purified by three solubilization-precipitation cycles using DMF and methanol as solvent and precipitant, respectively, then soaked in Milli-Q water for two days and dried in a vacuum oven at 120 °C until at a constant weight. Polymerization conversions of 70%, 80%, and 73% were obtained for PCNMA-RAFT, PSO_2_MA−RAFT1, and PSO_2_MA−RAFT2, respectively.

**Scheme 1:**
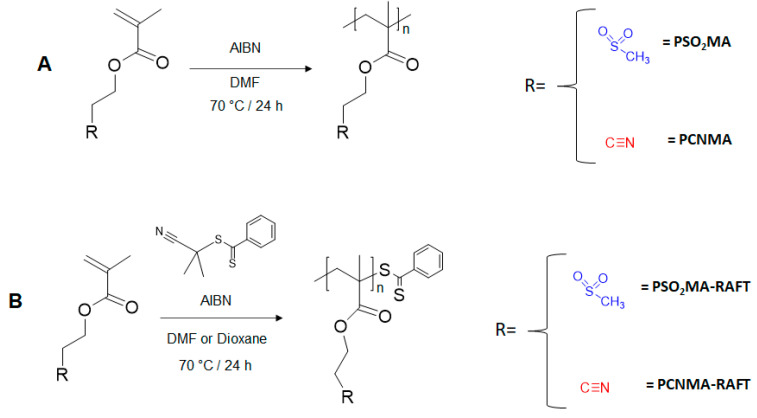
Conventional (**A**) and reversible addition-fragmentation chain-transfer polymerization (RAFT) (**B**) polymerization of SO_2_MA and CNMA monomers.

Scheme 1 represents both synthetic routes explained above.

### 2.4. Characterization Techniques

^1^H-NMR and ^13^C-NMR spectra were recorded at room temperature in a Bruker ACP-400 MHz NMR system using CDCl_3_ and DMSO-d_6_ for monomers and polymers, respectively. FTIR spectra of monomers and polymers were recorded in a Bruker Vector 22 spectrophotometer, between 600 and 4000 cm^−1^ after 34 scans in transmission mode. The molecular weight of polymers was determined by size-exclusion chromatography (SEC) using a Viscotek VE 1122 solvent delivery system with a VE 7510 GPC degasser and Viscotek VE 3508 differential refractive index (R.I.) detector coupled to three Viscotek LT4000L mixed columns. *N,N*-Dimethylformamide was used as eluent (0.5 mL/min flow rate) and PMMA standards were used for conventional calibration. Density of polymer films after dielectric measurements was calculated using a top-loading electronic METTLER balance (ME-33360) and diethyl ether (density 0.713 g/cm^3^ at room temperature). Density values for these polymethacrylates seem not to be affected by the differences in molecular weights and dispersity indexes obtained in the present work. Polymethacrylates with sulfonyl groups showed a density value of 1.39 g/cm^3^, while those containing nitriles exhibited a value of 1.22 g/cm^3^. Besides density values, refraction index of polymer films was measured using a Metricon Model 2010 Prism Coupler after dielectric measurements. Average-values of 1.5253 and 1.5022 were obtained for samples containing sulfonyl and nitrile entities, respectively.

Thermal behavior was studied using a TGA/SDTA851 Mettler-Toledo thermobalance (TGA) and Mettler-Toledo DSC 821e (DSC) previously calibrated by protocols established by Mettler-Toledo. TGA thermograms were recorded between 25 and 900 °C at 20 °C/min, while DSC analysis was performed following five steps: (i) From 25 to 200 °C with a heating rate of 20 °C/min; (ii) 5 min at 200 °C; (iii) from 200 to 0 °C at a cooling rate of 20 °C/min; (iv) 5 min at 0 °C and (v) from 0 to 200 °C at a heating rate of 10 °C/min. Glass transition temperatures (*T*_g_) were calculated from the inflection point measured in the last cycle using the STARe version 8.1 program from Mettler. Both TGA and DSC measurements were performed under N_2_ atmosphere (20 mL/min).

Broadband dielectric spectroscopy (BDS) measurements were carried out by a Novocontrol Alpha high-resolution analyzer. Polymer powders were placed between flat gold platted electrodes (20 mm diameter) in a sandwich configuration and hot-pressed at 130 °C. These capacitor-like configurations were placed in an oven pre-heated at 120 °C under vacuum overnight before starting dielectric measurements, allowing the formation of bubble-free films with an average thickness of 0.500 ± 0.005 mm. Isochronal scans were measured under inert N_2_ atmosphere and over a temperature range from −150 °C to 100 °C at different frequencies (from 1 Hz to 10^6^ Hz) using an AC voltage of 1.0 V. Using a QUATRO system from Novocontrol, the temperature of each experiment was controlled with an error of 0.1 °C.

## 3. Results and Discussion

### 3.1. Structural Characterization

**Figure 1:**
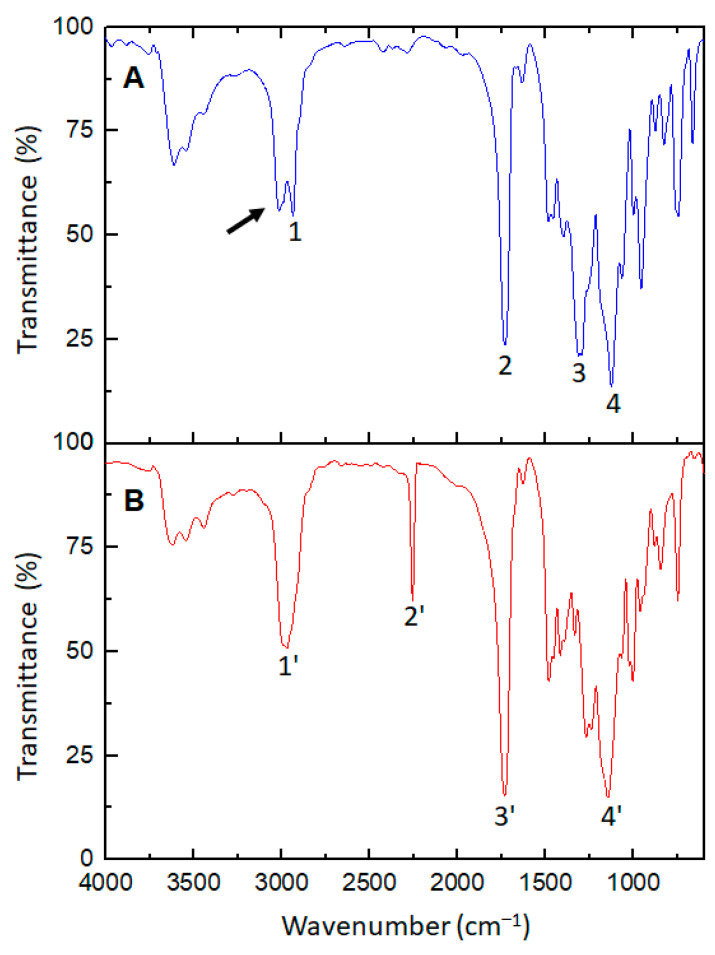
FTIR spectra of PSO_2_MA (**A**) and poly(2−cyanoethyl methacrylate) (PCNMA) (**B**).

The structural characterization was carried out in terms of FTIR and RMN spectroscopies. Figure 1 shows FTIR spectra of both polymethacrylates obtained by conventional radical polymerization, since as it was expected, identical spectra were recorded for those synthesized by RAFT (data not shown).

Both spectra confirmed the presence of sulfonyl and nitrile functional groups in the respective polymethacrylates, which are responsible for the outstanding dielectric properties reported by these materials. The presence of sulfonyl groups can be demonstrated from signals labeled as 3 and 4 in Figure 1A, centered at 1313 and 1127 cm^−1^, respectively, representing the asymmetric and symmetric vibrations of S–O double bonds [1,7,29]. Moreover, the signal pointed out by a black arrow (3013 cm^−1^) would be assigned to the vibration of the methyl group directly bonded to the sulfur atom [29]. On the other hand, Figure 1B reveals the presence of nitrile groups in PCNMA and PCNMA−RAFT through the signal 2′ centered at 2255 cm^−1^ [4,5,24,30]. Regardless of the above, other characteristic vibrational bands for polymethacrylates can be detected. Vibrational modes attributed to CH_2_ and CH_3_ (signals 1 and 1′) appear at 2950 cm^−1^, signals 2 and 3′ observed at 1730 cm^−1^ are related to the stretching of carbonyl groups, and finally, signal 4′ detected in Figure 1B represents the stretching mode of O–C–O bonds belonging to ester groups [1,7,31,32]. This last signal, centered at 1147 cm^−1^, cannot be observed in Figure 1A, probably due to its overlap with sulfone vibrational bands.

**Figure 2:**
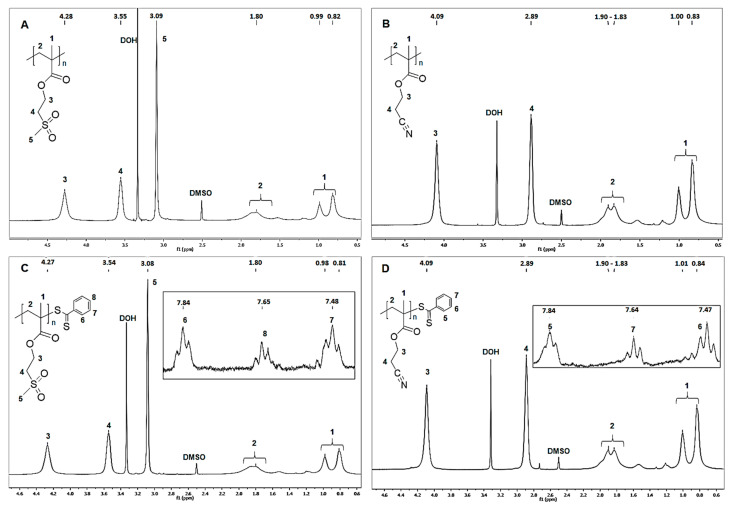
^1^H-NMR spectra of both polymethacrylates polymerized conventionally (**A**,**B**) and by RAFT methodology (**C**,**D**). DMSO-d_6_ used as solvent.

Figure 2 and Figure S1 (see Supplementary Materials) show ^1^H-NMR and ^13^C-NMR spectra, respectively, for polymethacrylates obtained by both polymerization techniques.

As shown in Figure 2, a complete signal assignation was successfully achieved. Moreover, contrary to FTIR, the NMR analysis allows the identification of certain differences between samples obtained by both polymerization methods. In particular, spectra recorded for samples synthesized by RAFT, exhibit typical signals related to the aromatic portion of the RAFT agent used (2CPBz). These signals and their chemical displacements have been previously reported [33] and would be indicative of the retention of the RAFT agent in the terminals of some polymer chains [27,28]. Similarly, Figure S1 reveals the apparition of signals in samples polymerized by RAFT, which are absent for samples synthesized through conventional radical polymerization. Additionally, it is important to mention that the shape shown by signals labeled as 1 and 2 in all ^1^H-NMR spectra would be due to the inherent tacticity exhibited by polymethacrylate systems [34]. The above argument could also serve as explanation for the multiple peaks assigned to signals A, B, C, and D in all ^13^C-NMR spectra [1,7]. This phenomenon has been broadly studied in previous works [19,34,35,36] from which, following its reported protocols, the tacticity of PSO_2_MA, PCNMA, and those polymerized by RAFT was calculated. Table 1 summarizes tacticity results obtained.

Table 1 shows that all these materials are mostly syndiotactic, presenting similar values regardless of the polymerization method. This result could be considered reasonable since both mechanisms are based on radical pathways, and therefore, the same propagating radical intermediates can be found during the propagation step. Regarding the above, Park et al. [37] found that poly(methylmethacrylate) with different tacticity exhibit variations in dielectric properties. Therefore, the tacticity values obtained for our materials should enable us to discard any effect on their dielectric behavior, allowing us to focus our discussion on the effects of molecular weight and dispersity values.

**Figure 3:**
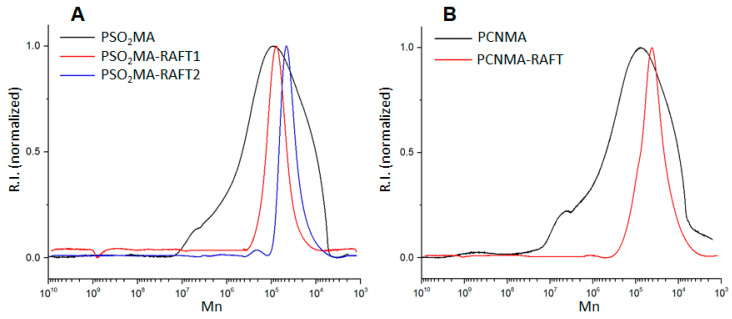
Size-exclusion chromatography (SEC) traces obtained for PSO_2_MA (**A**) and PCNMA (**B**) polymerized by both methodologies.

Molecular weight and dispersity values obtained by SEC. Results are shown in Figure 3 and summarized in Table 2.

SEC results confirm the effectiveness of RAFT process against conventional radical polymerization in terms of Đ values obtained. As shown in Figure 3, polymers prepared by RAFT exhibit remarkably narrower molecular weight distributions, characterized by Đ values below 1.25. This fact, along with the retention of the RAFT agent in polymer chains, allows us to conclude that both methacrylic monomers are compatible with the RAFT process. Notwithstanding the above, Đ values obtained for PSO_2_MA and PCNMA agree with those expected for conventional radical polymerizations [1,7]. From now on, the present work will focus on the thermal and dielectric properties showed by these materials and how these properties were affected or not by variations in their molecular weights and Đ values.

### 3.2. Thermal Characterization

**Figure 4:**
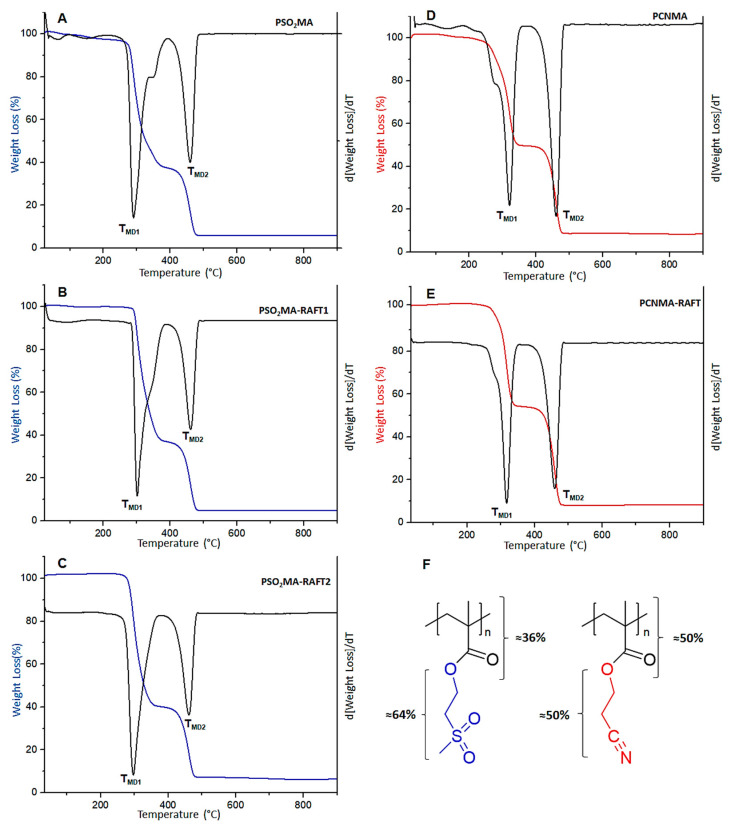
TGA and DTGA thermograms measured for all polymethacrylates containing sulfonyl (**A**–**C**) and nitrile (**D**,**E**) functional groups, along with the theoretical mass contribution calculated for their pendant groups and main chains (**F**).

Thermal stability of all polymers was tested by means of thermogravimetric analysis (TGA). Results can be seen in Figure 4, from which thermal parameters showed in Table 3 were calculated.

Firstly, all polymers were heated at 200 °C for 5 min, after which FTIR spectra were recorded again finding no significant changes with respect to the polymer before heating. Thereby, mass losses observed at this temperature interval could be related to the volatilization of low molecular weight compounds such as solvents, monomers, oligomer-like species, impurities, etc. Regarding the above, it draws attention that those polymers obtained through conventional polymerization evidence clear mass losses below 200 °C. This could be ascribed to the elimination of low molecular weight compounds present in these polymers exhibiting a considerable broader molecular weight distribution. This lower purity could be related to the higher amount of solvent used during the polymerization process, which not only facilitate the incorporation of typical impurities in samples (e.g., occluded solvent molecules, ions, etc.), but also could induce the occurrence of transfer reactions where radical species suffer an earlier stop in their growth. The above, together with the abundant termination reactions occurring in conventional radical polymerizations, would allow the existence of some of the aforementioned low molecular weight species. Thereby, under these conditions, RAFT polymerization seems to produce cleaner materials, which is a priority for dielectric applications.

In terms of thermal stability, all polymers obtained shown T_i_ values above 250 °C, allowing them to be considered as materials with adequate thermal resistance [38]. In addition, as a trend, it can be observed that polymethacrylates containing sulfonyl groups are slightly more stable, in terms of T_i_, than those with nitriles. Since thermal degradation mechanisms of polymers are still matters of discussion, it is difficult to propose a precise explanation about the effect that these functional groups exert over the thermal stability. However, a good model for understanding the process behind the decomposition of polymers corresponds to the visualization of a polymeric matrix being affected by successive heat waves coming from a heating source. When these waves reach the surface of the material, they are absorbed and, consequently, vibrational waves propagating along the matrix are produced. These waves travel over the material finding each other, from which constructive and destructive interference phenomena can occur. In this sense, when two or more waves participate in a constructive interference, and the sum of their energies exceeds energies of bonds present in the material, they will begin to break, fragmenting the material in a random way and generating reactive species (e.g., radicals, ions) which, under these high-temperature conditions, favor depolymerization processes [39,40].

**Figure 5:**
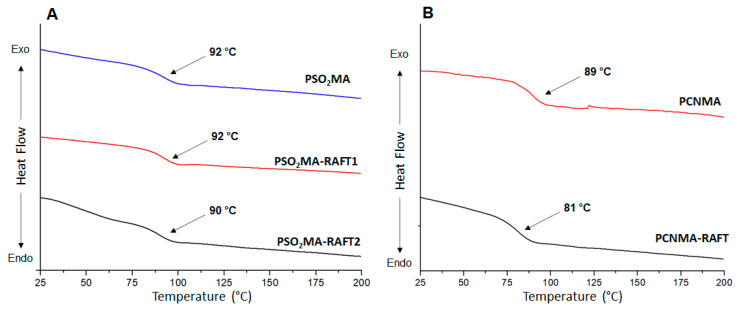
DSC analysis of polymethacrylates containing sulfonyl (**A**) and nitrile (**B**) groups. *T*_g_ values are indicated by black arrows.

Considering the above model, and limiting the discussion to polymethacrylate systems, the increased T_i_ values showed by polymers containing sulfonyl groups could be due to the higher dipole moment that these entities exhibit against nitriles. These dipolar groups should induce stronger dipole-dipole forces, allowing for obtaining materials with a more reinforced inner structure [29]. This reinforced matrix would be more tolerant to the thermally activated waves mentioned above, allowing the delay of the onset thermal degradation. Some experimental indications supporting the above would be the considerable higher density showed by PSO_2_MA polymers in comparison with PCNMA samples, and also their higher *T*_g_ values (see Figure 5). Both results would be indicative of polymers with a higher degree of packing and higher stiffness, properties that are usually ascribed to more resistant structures. Previous reports have already attributed high density values and increased glass transition temperatures to polymers containing high dipole moment functionalities [25,29]. Another interesting result is that, regardless the dipolar entity, those polymers prepared through RAFT polymerization show higher thermal stability. This result correlates well with previous works reporting a delay in the thermal decomposition with the decrease of molecular weight in polymers [41,42,43].

Concerning the shape of thermograms shown in Figure 4, it can be seen that all polymethacrylates, independently of their molecular weight and dispersity, exhibit a roughly two-step degradation process. The above has been analyzed and discussed by previous authors [1,29], including our group [7,20], assigning the first decomposition process mainly to the cleavage of pendant groups, while the second step would be ascribed to the volatilization of species arising from the random fragmentation and depolymerization of the polymer backbone remaining from the first step. For this first degradation step, the area analysis performed on DTGA curves reveals a mass loss percentage of around 61%, 63%, and 61% for PSO_2_MA, PSO_2_MA−RAFT1, and PSO_2_MA−RAFT2, respectively, whereas for PCNMA and PCNMA−RAFT values of 49% and 46% were calculated. These values show a good correlation with the mass percentage of pendant groups pointed out in Figure 4F. Furthermore, as our group has previously reported for both polymethacrylates, the FTIR analysis of residues obtained after the first decomposition presents the total absence of sulfonyl and nitrile bands [7]. In contrast, other bands assigned to the polymer main chain remain in spectra.

Figure 5 shows DSC measurements performed for all materials.

All polymethacrylates are amorphous materials, with *T*_g_ values above room temperature. Moreover, it draws attention that these values are well above than those reported for polymethacrylates containing propyl (≈35 °C) and butyl (≈19 °C) side groups [44], which can be considered analog structures without dipolar groups. Therefore, as was stated before, dipolar forces arisen from the presence of sulfonyl and nitrile groups are expected to increase the *T*_g_ values for these materials [29]. In addition, the higher bulkiness and dipole moment of sulfonyl entities could explain the higher *T*_g_ showed by polymers containing these functionalities. On the other hand, it is well-known that the glass transition phenomenon depends on different factors, one of them being the molecular weight of the polymer [19,45]. DSC results shown are in accordance with the typical behavior expected, with higher *T*_g_ values obtained for higher molecular weight samples. However, while for PCNMA and PCNMA−RAFT a considerable change in their *T*_g_ values is observed, this was not the case for those containing sulfonyl groups. This could be related to the Flory–Fox relation [19,46], which showed that for a given polymer there is a molecular weight value beyond which its *T*_g_ remains almost unaltered. Therefore, it could be possible that polymethacrylates with sulfonyl groups are close to this critical molecular weight predicted by the Flory–Fox relation. However, it is important to consider that to date there are not specific studies correlating *T*_g_ and Mn values for these specific polymers. The few works in which these materials appear in the bibliography show *T*_g_ values remarkably similar to those reported in this work, probably because the molecular weights fall by the same order of magnitude. Therefore, further studies are required to expand the range of molecular weights for both polymers, which would allow to carry out a more reliable adjust of the Flory–Fox relation to the thermal behavior of these materials. Finally, it is important to mention that since all *T*_g_ values are well above room temperature, these materials correctly meet the definition of *dipolar glass polymers.*

### 3.3. Dielectric Characterization

**Figure 6:**
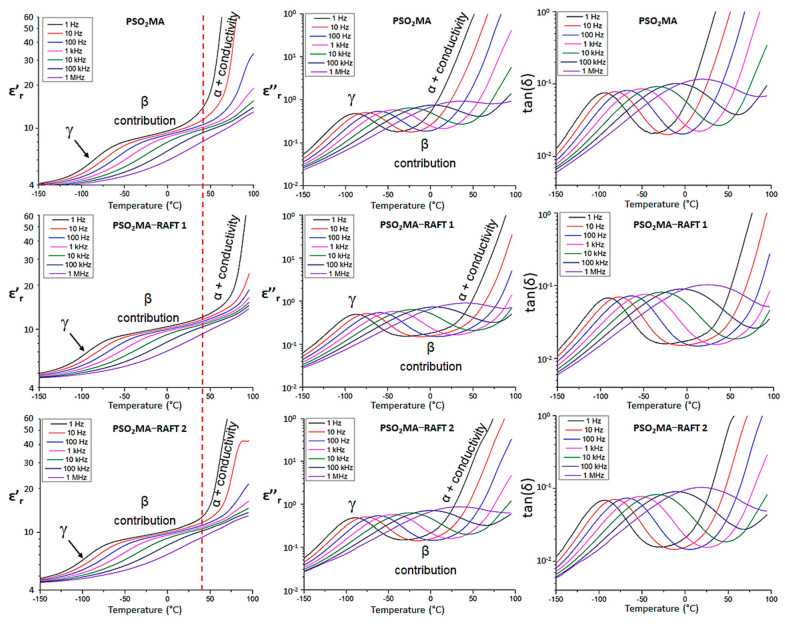
Evolution of ε’_r_, ε”_r_ and tan(δ) with the temperature at different frequencies for PSO_2_MA, PSO_2_MA−RAFT1, and PSO_2_MA−RAFT2.

As was mentioned before, the dielectric properties of both polymethacrylates, PSO_2_MA and PCNMA, have been studied in previous reports; firstly by Wei et al. [1] in their pioneering work about PSO_2_MA, the first *dipolar glass polymer* reported, and then by our own group [7], showing a complete study and comparison regarding dielectric properties of PSO_2_MA and, for the first time, PCNMA. In both works, polymers were synthesized by conventional radical polymerization, obtaining materials with a high dispersity and low reproducibility in terms of synthesis. The above situation has served as motivation for using more controlled and reproducible protocols, allowing the re-evaluation of dielectric properties for these materials. The dielectric characterization will start analyzing results exposed by sulfonyl-containing polymethacrylates, shown in Figure 6.

Figure 6 exhibits isochronal measurements of ε’_r_, ε”_r_, and tan(δ) for PSO_2_MA, PSO_2_MA−RAFT1, and PSO_2_MA−RAFT2, showing a remarkably similar behavior despite the polymerization method employed. For all these materials, their dielectric behavior in terms of ε’_r_ can be separated into three different regions, in which the evolution of this parameter exhibits a dissimilar behavior. The first one, identified from −150 °C to around −50 °C, is characterized by a clear step jump in the ε’_r_ value. Moreover, this rise of ε’_r_ occurs in the same temperature range at which a strong transition (or relaxation) in ε”_r_ curves is detected. This transition, labeled as γ, has been successfully attributed in previous reports to sulfonyl groups’ rotational motions and is responsible for the outstanding dielectric properties shown by these materials [1,7,20]. The second goes from −50 °C and temperatures slightly above room temperature, where a smooth but constant rise of ε’_r_ can be visualized, attributed to the participation of some remnant sulfonyl groups activated at higher temperatures, together with the activation of motions ascribed to ester functionalities which, based on previous reports [47,48], begin to contribute to the polarization of the material in this range of temperatures. The motion of the ester group in polymethacrylates is well-known as the β transition [48,49]. Finally, the third region starts at around 50 °C and reveals an abrupt increase of ε’_r_ as the temperature rises. This region has been labeled as “α + conductivity” indicating the merge of two different processes but arising from the same phenomenon, the glass transition. Regarding the above, at temperatures close and above *T*_g_, besides rotational motions of dipolar groups (γ and β), long-range motions of polymer chains appear, allowing the material to reach a higher polarized state due to translational movements [1,12]. These cooperative chain movements correspond to the well-known α relaxation, closely related to the glass transition of the system [49,50]. On the other hand, the second process emerges from the diffusion of ionic species due to the higher fluidity of the material at these temperatures. The greater mobility of ionic species, usually present in the form of impurities, induces a dramatic increase in the conductivity of the material [13], directly damaging energy storage properties of these materials, as can be seen from the abrupt increment of ε”_r_ and tan(δ) values. Additionally, besides the contribution of translational movements to the material polarization, the abrupt increase detected for ε’_r_ would also be related to the electrode-polarization phenomenon, understood as the accumulation of ionic species at the electrode-sample interface [12,50].

**Figure 7:**
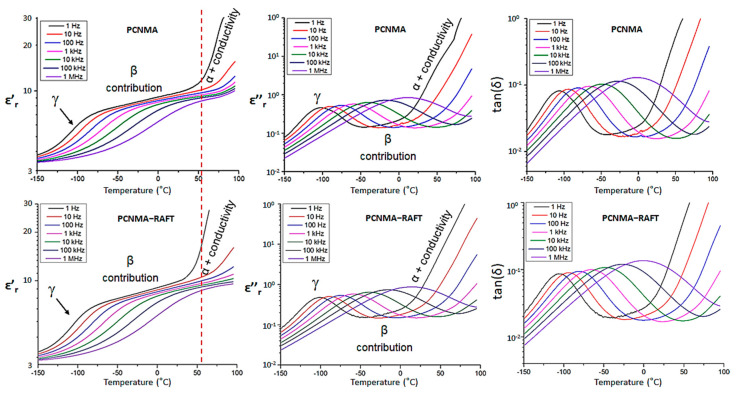
Evolution of ε’_r_, ε”_r_, and tan(δ) with the temperature at different frequencies for PCNMA and PCNMA−RAFT.

The previous analysis is also consistent with the dielectric results shown in Figure 7 for nitrile-containing polymethacrylates, in which their behavior can be understood following the above discussion. The same three regions can be detected, along with γ and β transitions assigned to local motions of nitriles and ester entities, respectively [7].

In general, since these polymers share similar structures except for those differences in terms of the dipolar group and molecular weight, they all exhibit a remarkably similar dielectric behavior. In fact, this behavior involves the occurrence of two consecutive states: The *dipolar glass polymer* state, observed during the first two regions mentioned above, and the *paraelectric* state, which starts to be present in the third region. The *dipolar glass polymer* state takes place at temperatures below *T*_g_, at which these amorphous polymers remain vitreous. The reason for the above is avoiding the presence of significant dissipative phenomena (e.g., α relaxation and ionic conductivity). Thereby, the polarization of the material is achieved by sub-*T*_g_ transitions ascribed to rotational motions of dipolar groups, allowing the obtention of polymers with high dielectric constants and relatively low loss factors. On the other hand, at temperatures above *T*_g_, the *paraelectric* state is achieved [1]. This state is characterized by a high polarization of the material, which is induced by the increased fluidity of the polymeric medium. This greater fluidity, probably from materials in rubbery state, allows to decrease the hindering of rotational motions of dipolar structures, along with adding the contribution of translational motions of polymer chains to the total polarization of the material [4,20]. Therefore, since a higher number of dipoles are able to align with the external electric field, a considerable rise in the dielectric constant value is achieved. However, as was mentioned before, the higher fluidity of the media also allows the appearance of the ionic conductivity, triggering a dramatic increase of tan(δ) values. Based on the above, it is clear that under these conditions, the *paraelectric* state is not suitable for energy storage applications and, therefore, the dielectric properties usually reported for these materials are measured from the *dipolar glass polymer* regime. Table 4 summarizes ε’_r_ and tan(δ) values recorded at 25 °C from isochronal measurements. The results shown below allow these polymers to be considered as dielectric materials with high dielectric constant and relatively low losses, exhibiting ε’_r_ values well above the average for conventional polymers [12,13].

**Figure 8:**
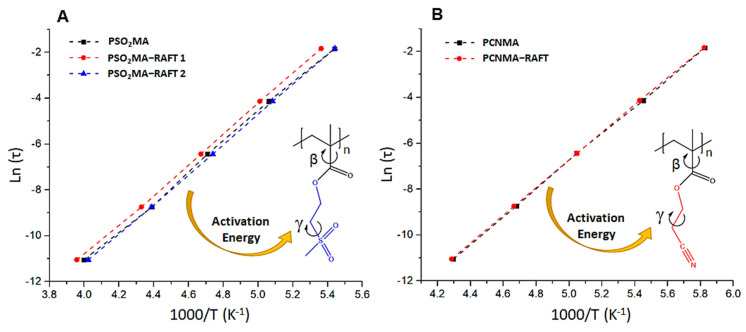
Arrhenius plots for polymethacrylates containing sulfonyl (**A**) and nitrile functional groups (**B**).

From Table 4, the superiority of sulfonyl-containing polymers in terms of ε’_r_ values is exposed, corroborating our previously reported results. At first glance, this could be attributed to the higher dipole moment of sulfonyl entities compared to nitriles; however, besides this factor, the density of dipoles and their mobility inside the material must be considered. This has already been studied by our group [7], finding that even when polymethacrylates containing sulfonyl groups present lower dipole densities, the higher dipole moment of sulfones and their greater mobility with respect to nitriles, allows these materials to exhibit a greater polarization. By using the refractive index together with the density of polymers obtained in this work (see Section 2.4), and following our protocol based on the Onsager equation [4,5,7,51], it is possible to successfully corroborate our previous calculations. Another interesting result deduced from Table 4, arises from the fact that for polymers containing the same dipolar groups, but different molecular weights and dispersity, almost identical values of ε’_r_ and tan(δ) were obtained. Moreover, these values are in total accordance with those previously obtained by Wei et al. [1] and by our group [7], allowing to include in this analysis three new samples with different molecular weight and dispersity values. The previous result was already expected considering that all these materials, regardless of their molecular weights, did not show variation in their density and refractive index. Thereby, since the dielectric performance of these materials is governed mainly by the local movements of sulfonyl and nitrile structures, the fact that the density remains unchanged allows us to believe that the local environment that surrounds these structures is not significantly modified by altering their molecular weight. In order to corroborate the previous idea, as an approximation, activation energies (Ea) of sulfones and nitriles motions (γ transitions) were calculated from isochronal measurements (Figure 6 and Figure 7). To achieve this, the Arrhenius formalism [51] (Equation (1)) was applied by fitting the temperature dependence of the relaxation time ($t_{\max}$) obtained from the maximum of γ transitions recorded at different frequencies ($f_{\max}$). Results are shown in Figure 8.

(1) $$\ln(t_{\max})=\ln(t_{\infty })+\frac{Ea}{K_{B}T}\;;\;\mathrm{with}\;t_{\max}=\frac{1}{2\pi f_{\max}}$$

The activation energy of γ transitions represent the energy barrier that sulfones or nitriles, need to overpass to change their orientation and contribute to the polarization of the material. Besides, the magnitude of this barrier should be directly influenced by the surrounding media of dipoles. In this sense, for a same type of dipolar group, any change in the measured value of its activation energy could be attributed to variations in its local environment. From the slopes of the linear fits exposed in Figure 8A, Ea values of 0.56, 0.57, and 0.56 eV were calculated for sulfones movements in PSO_2_MA, PSO_2_MA-RAFT1, and PSO_2_MA-RAFT2, respectively. Similarly, from Figure 8B, nitrile motions in both PCNMA and PCNMA-RAFT exhibit Ea values of 0.52 eV. The previous result suggests that, within the explored range, changes in the molecular weight of these polymers do not alter the energy barrier, and consequently, the kinetic behavior of sulfones and nitriles motions. Thereby, since the dielectric constant of obtained materials depends on these dipolar motions, but these motions are not affected by changes in their molecular weights, values in Table 4 find a plausible explanation. Additionally, it is important to mention that Ea values calculated in this work are in good correlation with those previously reported [1,7]. Higher values have been again obtained for sulfonyl-containing polymethacrylates. This has previously been attributed to the stronger dipole–dipole interactions and greater steric hindrance that sulfone groups exhibit regarding nitriles. Lower Ea values mean that dipole rotations require lower thermal energy to be activated. Thereby, since nitrile groups start to move at lower temperatures than sulfones (see Table 5), polymethacrylates containing the first entities will reach a polarized state earlier on the temperature scale.

On the other hand, contrary to what has been discussed so far, a more detailed analysis of Figure 6 and Figure 7 in the high-temperature regime reveals differences in the dielectric behavior of these materials, that is to say, where the *paraelectric* state begins to be present. The red dotted line attached to the ε’_r_ profiles, helps to visualize the shifts in the temperature scale where α relaxations and ionic conductivity phenomena begin to occur for each polymer. Since the molecular weights and dispersity values are the main differences ascribed to these polymers, the advance or delay on the temperature scale of the paraelectric state must be related to both properties. In fact, the close relationship between the α relaxation, ionic conductivity, paraelectric state, and glass transition temperature has been widely confirmed [10,12,13,38]. From isochronal measurements recorded at 1 Hz, the temperatures at which γ transitions and the *paraelectric* state appear were obtained. The results for each polymer are summarized in Table 5.

The analysis of the results presented in Table 5 will begin focusing on polymethacrylates containing sulfonyl groups. Usually, since polymers with high molecular weight tend to exhibit increased *T*_g_, it is highly expected to observe a delay on the temperature scale for the rise of the paraelectric state. However, it draws attention that even when PSO_2_MA presents the highest Mn and share the same *T*_g_ value with PSO_2_MA-RAFT1, it shows the lowest temperature for the onset of the *paraelectric* state. A first explanation could be related to the presence of a greater quantity of impurities, allowing earlier detection of the ionic conductivity by the equipment. However, a second possible argument would be related to the notably higher Đ value shown by this polymer. In this sense, the presence of numerous fractions with different molecular weight inside the sample, could also generate heterogeneity in terms of thermally activated long-range motions, where some of these movements could be detected at lower temperatures. It worth noting that both previous arguments would also serve as explanation to the slightly higher ε’_r_ and tan(δ) values measured at for PSO_2_MA. On the other hand, when comparing materials with similar Đ values, as PSO_2_MA-RAFT1 and PSO_2_MA-RAFT2, the one with the higher Mn value will present a more delayed *paraelectric* state and, consequently, a less dissipative behavior over the same temperature range. Therefore, as can be seen in Table 5, PSO_2_MA-RAFT1 behaves as a *dipolar glass polymer* in a wider temperature range, reaching the highest working temperature among the others. Moreover, it worth noting that even when the molecular weight of PSO_2_MA-RAFT2 is considerably lower than that measured for PSO_2_MA, the magnitude of their working temperature ranges is remarkably similar. Finally, referring to nitrile-containing polymethacrylates, it was found that the *paraelectric* state in PCNMA is achieved at temperatures slightly above that of PCNMA-RAFT. This is a remarkable result, taking into account the considerable higher *T*_g_ value of PCNMA. Similar to PSO_2_MA, it could be attributed to the wider molecular weight distribution that broadens the temperature range where long-range motions start to be active. In this regard, it is expected that PCNMA having the same molecular weight but a substantial lower Đ value, exhibits a notably increase of dipolar glass polymer range. Furthermore, we believe that designing a PCNMA-RAFT with a Mn closer to PCNMA would result in the obtention of a material with an effective delay of dissipative phenomena to a similar extent to the one observed for polymethacrylates bearing sulfonyl entities. Further studies will be carried out in the near future aiming to corroborate these hypotheses.

In summary, the variations of molecular weights and dispersity indexes within the explored range of these polymeric materials do not seem to significantly affect their polarization capacity, defined by ε’_r_, ε”_r_, and tan(δ). However, the increase in their molecular weight while keeping the dispersity values low, allows extending the outstanding dielectric properties of these polymethacrylates towards higher temperatures, managing to broaden the working temperature range by delaying the appearance of well-known dissipative phenomena.

## 4. Conclusions

Polymethacrylates containing sulfone and nitrile groups were successfully synthesized by conventional radical polymerization and by RAFT, obtaining materials with notable differences in terms of their molecular weights and dispersities. Their chemical structures and molecular weights have been probed in terms of FTIR, NMR, and SEC analysis.

TGA and DSC results showed that all polymers are amorphous materials with glass transitions above room temperature and presenting adequate thermal resistance, since all of them degrades above 250 °C. Polymers containing sulfonyl entities exhibit higher T_i_ and *T*_g_ values, which could be related to the strongest interactions generated by this group and its effect on the internal structure of these materials.

Regarding dielectric properties, all polymers showed dielectric constant values above 9 and loss factors around-below 0.02, allowing it to be considered as high dielectric constant materials with a relatively low dissipative behavior. Due to the above, these materials are suitable for capacitor applications. However, the sulfonyl group was shown to produce materials with higher ε’_r_ values, attributed to its higher dipole moment and higher mobility within the polymer matrix. On the other hand, considering the explored range, since variations on the molecular weight and dispersity in these materials shown no effect on the sulfones and nitriles behavior (revealed by Ea calculations), parameters ε’_r_, ε”_r_, and tan (δ) seem not to be affected by these properties, at least in the studied range. Notwithstanding the above, the present work revealed a notable effect on the delay of the paraelectric state when modifying the values of Mn and Đ, allowing to avoid earlier apparition of highly dissipative phenomena. Based on the above, the synthesis of dipolar glass polymers with well-managed dispersion and molecular weight values allows expanding their dielectric properties towards higher temperatures, increasing the range of working temperatures where these materials exhibit, at the same time, a high polarization and low energy dissipation.

Lastly, it is well-known that polymers with increased molecular weights exhibit higher values of dielectric breakdown strength [50,51,52]. Therefore, complementing the above with our results, through the preparation of *dipolar glass polymers* of high molecular weight and low dispersity values, dielectric materials with better tolerance to high temperatures and voltages could be achieved, allowing to considerably broaden the spectrum of applications of these materials.

## Figures and Tables

**Table 1:** Tacticity values (%) of obtained polymethacrylates.

	PSO_2_MA	PSO_2_MA−RAFT1	PSO_2_MA−RAFT2	PCNMA	PCNMA−RAFT
Syndiotactic	62	57	59	62	64
atactic	35	37	35	34	33
isotactic	3	6	6	4	3

**Table 2:** Number-average molecular weight (Mn) and dispersity (Đ) values of synthesized polymers.

Sample	Mn (g/mol)	Đ (Mw/Mn)
PSO_2_MA	1.23 × 10^5^	2.18
PSO_2_MA−RAFT1	7.10 × 10^4^	1.18
PSO_2_MA−RAFT2	3.94 × 10^4^	1.12
PCNMA	1.13 × 10^5^	2.36
PCNMA−RAFT	3.25 × 10^4^	1.23

**Table 3:** Thermal degradation onset (T_i_), maximum weight loss rate temperature (T_MD_) and residue percentage (R) for PSO_2_MA and PCNMA prepared by RAFT and conventional radical polymerization.

Sample	T_i_ (°C)	T_MD1_ (°C)	T_MD2_ (°C)	Residue (%)
PSO_2_MA	280	291	460	5.8
PSO_2_MA−RAFT1	295	301	461	4.9
PSO_2_MA−RAFT2	283	297	461	6.4
PCNMA	265	320	460	8.5
PCNMA−RAFT	282	317	461	8.4

**Table 4:** ε’_r_ and tan(δ) values measured at 25 °C for polymethacrylates prepared by conventional radical polymerization and RAFT.

Sample	ε’_r_ (1 Hz)	ε’_r_ (1 kHz)	tan(δ) (1 kHz)
PSO_2_MA	11.9	10.0	0.020
PSO_2_MA−RAFT1	11.3	10.3	0.016
PSO_2_MA−RAFT2	11.3	10.5	0.016
PCNMA	9.8	9.0	0.016
PCNMA−RAFT	9.9	8.9	0.017

**Table 5:** Dipolar glass polymer temperature range determined for polymethacrylates obtained conventionally and by RAFT.

Sample	γ Transition Temperature (°C) ^a^	Onset Temperature of *Paraelectric* State (°C) ^b^	*Dipolar Glass Polymer* Temperature Range (°C) ^c^
PSO_2_MA	−89	48	137
PSO_2_MA-RAFT1	−87	76	163
PSO_2_MA-RAFT2	−89	49	138
PCNMA	−101	47	148
PCNMA-RAFT	−101	42	138

^a^ Values obtained from the maxima of peaks assigned to the relaxations of nitriles and sulfones in ε”_r_ isochronal plots measured at 1 Hz. ^b^ Values obtained from ε’_r_ isochronal plots measured at 1 Hz according to reference [20]. ^c^ These values represent the magnitude of the interval in which each material behaves as a dipolar glass polymer, calculated as (Onset temperature of paraelectric state)—(γ relaxation temperature).

## Data Availability

The data presented in this study are available on request from the corresponding author.

## Supplementary Materials

The following are available online at https://www.mdpi.com/2073-4360/13/3/317/s1, Figure S1: 13C-NMR spectra of both polymethacrylates polymerized conventionally (A,B) and by RAFT methodology (C,D). DMSO-d6 used as solvent.
