# Supplementary material for: Molecular Weight Enables Fine-Tuning the Thermal and Dielectric Properties of Polymethacrylates Bearing Sulfonyl and Nitrile Groups as Dipolar Entities
Source: Polymers (Basel). 2021 Jan 20;13(3):317. doi: 10.3390/polym13030317 (PMC7863962; doi:10.3390/polym13030317)
Supplement: Supplementary file 1 [file polymers-13-00317-s001.pdf]

Supporting Information

Molecular Weight Enables Fine-Tuning the Thermal and Dielectric Properties of Polymethacrylates Bearing Sulfonyl and Nitrile Groups as Dipolar Entities

Sebastian Bonardd [1,*], Cesar Saldías [2], Ángel Leiva [2], David Díaz Díaz [3,4,5] and Galder Kortaberria [6]

[1] Facultad de Ciencias, Centro de Nanotecnología Aplicada, Universidad Mayor, Camino la Pirámide 5750, Santiago 8580745, Chile
[2] Departamento de Química Física, Facultad de Química y de Farmacia, Pontificia Universidad Católica de Chile, Santiago 7820436, Chile; casaldia@uc.cl (C.S.); aleivac@uc.cl (Á.L.)
[3] Departamento de Química Orgánica, Universidad de La Laguna, Avda, Astrofísico Francisco Sánchez S/N, 38206 La Laguna, Tenerife, Spain; ddiazdiaz@ull.edu.es
[4] Instituto Universitario de Bio-Orgánica Antonio González, Universidad de La Laguna, Avda, Astrofísico Francisco Sánchez 2, 38206 La Laguna, Tenerife, Spain
[5] Institut für Organische Chemie, Universität Regensburg, Universitätsstr. 31, 93053 Regensburg, Germany
[6] Universidad País Vasco/Euskal Herriko Unibertsitatea, 'Materials + Technologies' Group, Dpto. Ingeniería Química y Medio Ambiente, Escuela Univ Politécnica, Pza Europa 1, 20018 Donostia-San Sebastián, Gipuzkoa, Spain; galder.cortaberria@ehu.es
* Correspondence: sebastian.bonardd@umayor.cl

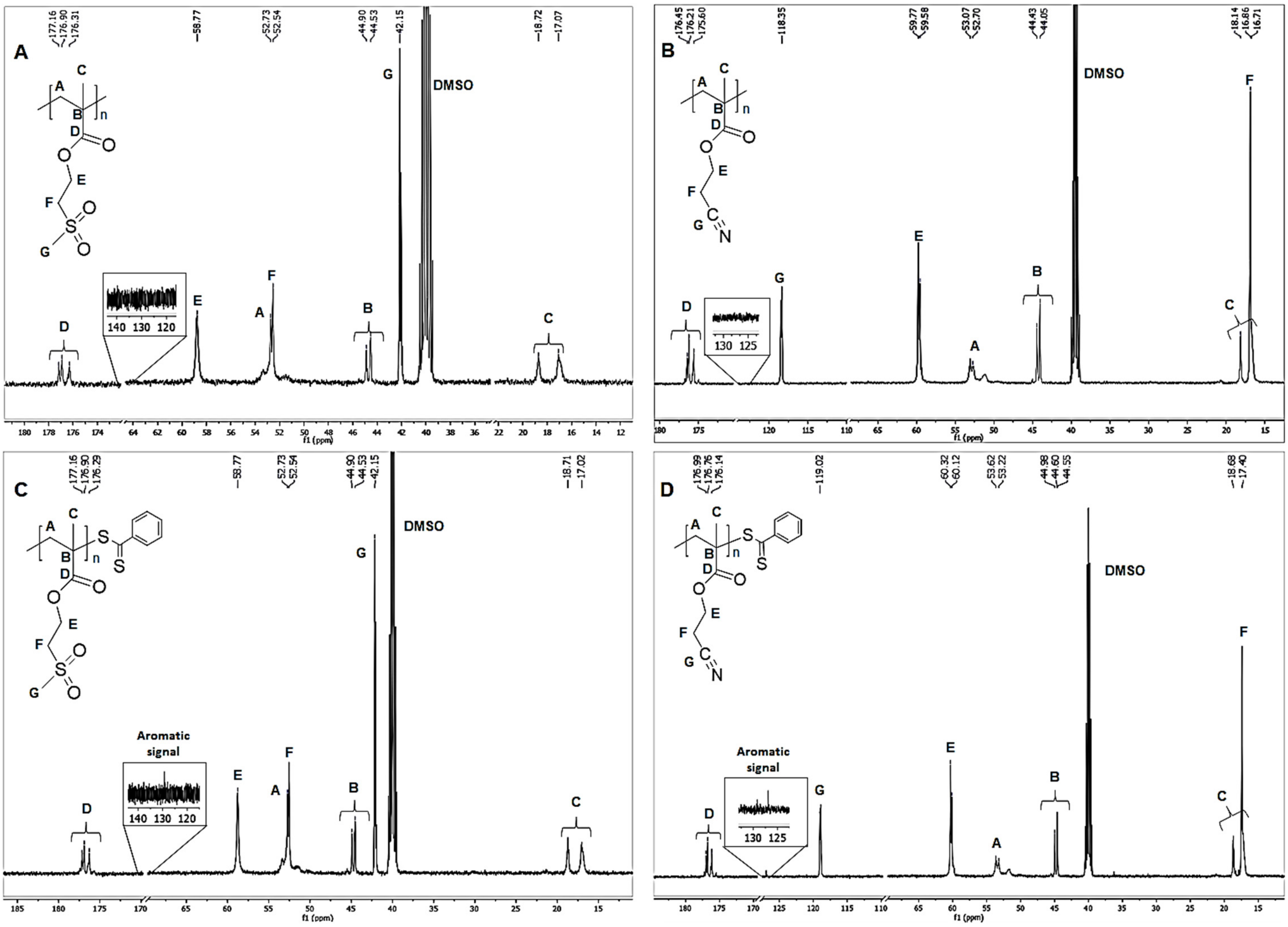

Figure S.1. ^{13}C-NMR spectra of both polymethacrylates polymerized conventionally (A, B) and by RAFT methodology (C, D). DMSO-d_6 used as solvent.
